# Risk factors analysis and prediction models of obesity in college students based on dietary patterns

**DOI:** 10.3389/fnut.2025.1598946

**Published:** 2025-09-11

**Authors:** Jiawang Bai, Mengyuan Chen, Wenfeng Hou, Yan Han, Jihong Shao, Ying Zhang, Yang Jiao, Hui Hua, Xiangmei Ren

**Affiliations:** ^1^School of Public Health, Xuzhou Medical University, Xuzhou, Jiangsu, China; ^2^Jiangsu Engineering Research Center of Biological Data Mining and Healthcare Transformation, Xuzhou Medical University, Xuzhou, Jiangsu, China

**Keywords:** risk prediction study, obesity, college students, dietary patterns, PBF

## Abstract

**Background:**

Overweight and obesity among college students have become significant public health concerns. This study aims to develop a nomogram model for assessing obesity risk in college students.

**Methods:**

A cross-sectional study was conducted among college students in Xuzhou. Demographic, dietary, and lifestyle information was obtained through self-administered questionnaires, while body composition was assessed using the InBody 570 analyzer. Dietary patterns and obesity prevalence were examined through multiple indicators. Principal component analysis (PCA), logistic regression, and a non-invasive risk assessment model based on percentage of body fat (PBF) were applied.

**Results:**

The vegetable meat grain dietary pattern and milk egg dietary pattern were associated with a reduced risk of PBF (*P* < 0.01), while the snack mode dietary pattern and aquatic meat dietary pattern increased the risk of PBF (*P* < 0.05). Binary logistic regression identified gender, physical activity, late-night snacking, regular meals, and a healthy diet as key predictors of PBF obesity in college students. The model achieved an area under curve (AUC) of 0.805, with a non-significant Hosmer–Lemeshow (H–L) test (*P* > 0.05). Decision curve analysis (DCA) showed that the model outperformed extreme curves, indicating its reliability.

**Conclusion:**

This study highlights the high prevalence of overweight and obesity among college students and the importance of using multiple indicators for comprehensive evaluation. The developed PBF-based nomogram model demonstrates potential for obesity screening but requires further validation in diverse populations.

## 1 Introduction

The global burden of overweight and obesity has tripled over the past 50 years, posing an urgent public health challenge (1). More than half the European and North American population was overweight or obese (2). Such trend is mirrored in China, where the prevalence of overweight and obesity among adults has exceeded 50% and is projected to reach 65.3% by 2030, with substantial implications for healthcare costs. This trend was expected to lead to medical costs exceeding 58.52 billion United States Dollar (USD), representing approximately 21.5% of China’s total healthcare expenditure (3). Beyond economic burdens, obesity profoundly compromises cognitive function through reduced concentration and memory linked to poor sleep, and psychological wellbeing by elevating depression and anxiety from weight stigmatization (4); manifesting as elevated clinical depression and anxiety disorders among obese students (5), pathological eating behaviors like binge-restrict cycles, and social withdrawal due to body image dissatisfaction, collectively impairing identity formation during this critical developmental stage and long-term health trajectories. Among college students, 47.3% with late-night snacking habits face 9.8-fold higher obesity risk, while high-fat dietary patterns like aquatic meat directly increase visceral adiposity. These synergistically impair academic performance, social adaptability, and lifelong productivity, underscoring the need for targeted interventions.

The prevalence of overweight and obesity was substantially high among college students (6). Several studies have shown that 1/3 college students have overweight or obesity and the prevalence rate of obesity in men was significantly higher than that in women (7, 8). Obesity is influenced by a complex interplay of genetic, environmental, and behavioral factors. While genetic predisposition plays a role, the rapid increase in obesity rates highlights the critical influence of environmental and lifestyle changes (9). Dietary habits and lifestyle factors may determine the obesity phenotypes in children and adolescents (10). Recent researches have linked overweight and obesity in this group to unhealthy habits such as a strong preference for high-fat and high-sugar foods, insufficient physical activity, inadequate sleep, and cigarette consumption (11–13). Furthermore, sociodemographic factors, including age, education level, household income, and nutritional knowledge, also contribute to the incidence of overweight and obesity (14).

There are various methods for measuring obesity, and different countries and regions may use different measurement indicators. Most studies only used a single indicator (15), while the combination of multiple indicators can comprehensively consider multiple factors, reduce errors, and improve prediction accuracy. The bioelectrical impedance analyzer (BIA) is a portable, efficient, and fast method that can simultaneously measure multiple obesity indicators, such as commonly used body mass index (BMI), PBF, visceral fat level (VFL), waist hip ratio (WHR), waist circumference (WC), and fat mass index (FMI) (16). Among these, PBF represents the proportion of body fat to total body weight, reflecting how much fat is present in the body. In some populations, although their BMI are within the normal range, their PBF may exceed the normal level and lead to hidden obesity. Abnormally high PBF increases the risk of related diseases such as high blood sugar (17). Therefore, PBF is more valuable as a reference for assessing individual health risks compared to other indicators.

Multiple studies have shown that obesity is closely related to diet, dietary factors greatly affect body composition, and dietary pattern analysis could comprehensively explore the impact of food intake on human health (18). Numerous studies have shown that dietary patterns are closely related to non communicable chronic diseases such as obesity, hypertension, and cardiovascular and cerebrovascular diseases. For example, a healthy dietary pattern is positively correlated with good levels of cardiorespiratory health. Numerous studies have demonstrated significant associations between specific dietary patterns and obesity risk in young adults. For instance, research among college populations indicates that inadequate vegetable consumption doubles obesity risk, while high intake of processed snacks correlates with higher visceral adiposity. Our study builds on this evidence by identifying four distinct dietary patterns in Chinese college students, including the obesity-promoting snack mode and protective vegetable meat grain patterns, quantifying population-specific risk (19, 20). Poor diet could become one of the main causes of overweight and obesity in adolescents (21).

Health risk assessment models played a crucial role in identifying individuals at higher risk of obesity and guiding targeted interventions (22). Classic models usually included BMI and Box-Cox power exponential (BCPE) model to assess obesity risk (23). However, important behavioral modifiable factors such as smoking, physical activity, and diet are often overlooked in these models. Therefore, developing a non-invasive, behavior-based risk assessment model that incorporates modifiable lifestyle factors was essential to enable the early and individualized interventions suggestions for college students at risk of obesity (24).

Hence, this study aimed to investigate the distribution and epidemiological characteristics of overweight and obesity among college students in Xuzhou, and to develop risk assessment models for different obesity types with visualization, providing a theoretical basis for obesity prevention and its practical application.

## 2 Materials and methods

### 2.1 Study design and setting

This was a cross-sectional study conducted among university students between October 2023 and June 2024 in Xuzhou, China. Participants were selected using a simple random sampling method, and both questionnaire and body composition measurements were employed to collect data through face-to-face interviews. The study adhered to ethical guidelines, and written informed consent was obtained from all participants prior to data collection. Ethical approval for this study was granted by the ethics committee (Approval No. XZFY 2024-052K-01J).

### 2.2 Study population

The target population consisted of undergraduate students aged 18–25 enrolled at university in Xuzhou. The current study recruited participants who fulfilled the following inclusion criteria: being physically healthy and between the ages of 18 and 25, spanning from freshmen to seniors. Exclusion criteria comprised: (1) chronic diseases affecting metabolic function, such as chronic wasting disease, chronic digestive tract disease, or thyroid/pituitary dysfunction; (2) conditions interfering with body composition measurements, including implantable electronic devices and metal implants.

### 2.3 Sample size and methods

The sample size was calculated using Epi Info 7.2.5.0, assuming 50% overweight and obesity prevalence to maximizing variability, 5% margin of error, and 95% confidence level, yielding a minimum of 385 participants. With a 35% non-response buffer, the target sample was 520. Ultimately, 1,080 students completed questionnaires; after exclusions, 885 were analyzed, exceeding the minimum requirement.

The fundamental formula for sample size calculation is used to estimate the prevalence of a single proportion such as the prevalence of overweight and obesity. The formula is as follows and verification has been performed:


$$n=\frac{Z^{2}\times p\times \left(1-p\right)}{E^{2}}$$

n: Minimum sample size (unadjusted). Z: The *Z*-value of the standard normal distribution corresponds to the confidence level. p: The estimated prevalence rate. E: The acceptable margin of error.

### 2.4 Data collection tools

A self-administered anonymous questionnaire of five sections was created to collect data after obtaining their consent for participation in the study. The first section collected the sociodemographic and health-related condition data including age, gender, grade, ethnicity, and family income. The second section collected data about lifestyle factors including smoking, drinking, exercise, prolonged sitting, mobile phone usage, length of sleep, and quality of sleep. The third section collected data on dietary behavior including breakfast, midnight snack, regular meals, number of meals, water, takeaway, and nutritional knowledge. The fourth section included the Food Frequency Questionnaire (FFQ) that collected the average weekly frequency of intake of 16 food items in the last three months, categorized based on the Chinese food composition table. The reliability and validity of the FFQ were assessed through a test-retest process. The two FFQ surveys were administered at a 1-month interval, yielding 25 pairs of complete questionnaires. These questionnaires, using 146 food items as a benchmark, were used to assess dietary factor score consistency. The FFQ reliability was confirmed with a Pearson correlation coefficient greater than 0.7. The final fifth section was the body composition measurement where researchers used BIA system, which provided detailed information on body weight, BMI, PBF, WHR, skeletal muscle mass, and total body water. Measurements were conducted in a controlled laboratory setting, with participants required to fast for at least four hours and empty their bladders prior to testing. All measurements were conducted by trained personnel to ensure accuracy and consistency.

### 2.5 Data collection plan

A pilot study was conducted to assess the feasibility, clarity, response rate, and completion time of the questionnaire. Based on the piloted population feedback some minor edits were made to improve the flow and comprehensibility of the questions. The questionnaire needed 5 to 10 min to be completed. Furthermore, all participants involved in the pilot study were excluded from the final analysis. Participants were invited to complete the questionnaire and FFQ on campus, and body composition measurements were taken immediately afterward. Before taking body the measurements, participants were asked to empty their stomachs, empty their bladders, remove their shoes and socks, stand in the designated area of the instrument, and place their front feet on the front foot electrodes and their heels on the back foot electrodes. During the measurement, participants placed their palms on the palm electrode and pressed their thumbs against the thumb electrode to touch the current. The analyzer automatically obtains body composition data such as BMI, PBF, VFL, WC, FMI, protein, skeletal muscle content, minerals, and total water content.

### 2.6 Dietary pattern and scoring

Principal component analysis (PCA) was used to identify underlying dietary patterns from the collected FFQ data. The Kaiser–Meyer–Olkin (KMO) test and Bartlett’s test of sphericity confirmed the suitability of the data for PCA. Factor loadings greater than or equal to 0.3 were considered significant and included in the scoring calculation. Participants were categorized into tertiles (T1, T2, T3) based on their adherence to specific dietary patterns. T3 represented the group most aligned with a given dietary pattern, which was used for further analysis. Further, a confirmatory analysis was used to extract different sample sizes for principal component analysis of dietary patterns to see whether the results were consistent.

Food items with absolute factor loading values ≥ 0.4 and non-repetitive in the extracted dietary patterns were selected as food groups for calculating the healthy dietary pattern score. According to the relationship between positive and negative effects of food items and obesity, the weight of food items was set to 1 and −1, respectively, and then the healthy dietary pattern score was constructed. The weight of 1 food group is a total of 7 items, namely, rice noodles, coarse grains, vegetables, fungi, dairy products, eggs, and soy products; the weight of −1 food group is a total of 7 items, respectively, pork, beef and mutton, poultry, aquatic products, oil products, desserts, snacks, beverages. The total score was calculated by assigning 1–4 points to the quartile of the daily intake frequency of each food item. The healthy dietary pattern not only preserves the dietary habits of participants inclined toward a healthy dietary pattern but also includes specific food items that influence obesity. A higher score in this dietary pattern indicates a stronger tendency toward a healthy dietary pattern. Additionally, a validation analysis was conducted in which the same participants completed the dietary questionnaire again after 2–4 weeks to assess the consistency of the scores between the two time points.

### 2.7 Risk assessment model

A risk assessment model for different types of obesity was developed using a combination of logistic regression and dietary pattern data. The dataset was randomly divided into training (70%) and validation (30%) groups. Logistic step-up regression analysis was applied to identify significant predictors of obesity. The final model was visualized using a nomogram, which provides an individualized risk score for obesity based on the identified factors. Model performance was evaluated using the area under the receiver operating characteristic (ROC), with a target AUC of ≥ 0.7 indicating acceptable predictive accuracy. Based on this, we build a risk assessment model for different types of obesity and display it visually, which provides a theoretical basis for obesity prevention work and can be applied in practice.

### 2.8 Statistical analysis

The data were collected, reviewed, and then fed to SPSS version 23. Numerical variables were described by the mean and standard deviation (SD), whereas categorical variables were described by number and percentage (%). A chi-square test was used to assess the association between the body fat layering variable and demographic and lifestyle variables including differences between males and females. Logistic regression analysis was used to analyze the relationship between different obesity factors, dietary patterns, and different types of obesity while adjusted odds ratios (ORs) and 95% confidence intervals (CIs) were calculated. A *p*-value < 0.05 was considered statistically significant.

## 3 Results

### 3.1 Baseline characteristics stratified by percentage of body fat

A total of 1,080 participants completed the questionnaire on diet and lifestyle habits, of which 945 volunteered to provide a weekly food frequency questionnaire and body composition measurement, among which 5 participants did not meet the requirements of the measurement of the composition of the body. When sorting out the data, it was found that 55 participants filled in the questionnaire incompletely or repeatedly, and 885 students were finally included in the study. Among them, there were 392 (44.3%) females and 493 (55.7%) males. 412 (46.5%) of participants were 20 years old or younger and 600 (67.8%) had a low level of income. The overall characteristics of the participants, based on PBF classification (25–28), are presented in Table 1. There was a significant difference in the number of obese and overweight individuals between males and females (χ^2^ = 44.256, *p* < 0.01), with men more likely to be obese or overweight than women. Alcohol consumption showed a significant association with obesity (χ^2^ = 16.907, *p* = 0.001), with 49.2% of overweight participants reporting alcohol consumption compared to 39.9% of normal-weight participants. Physical exercise showed a significant association with obesity (χ^2^ = 43.465, *p* < 0.001), with 49.9% of inactive individuals being overweight or obese compared to 29.1% obesity among the active group. A significant difference was observed in water intake (χ^2^ = 16.137, *p* < 0.001), with 56.4% of participants consuming less than 2 liters per day being overweight or obese compared to 40.7% prevalence of obesity among participants consuming more than 1 liter.

**TABLE 1:** Baseline characteristics stratified by percentage of body fat.

Variables	PBF (%)			*P*
	Low	Normal	High	
**—Gender, *n* (%)**				
Females	69 (17.6)	195 (49.7)	128 (32.7)	< 0.01**
Males	53 (10.8)	169 (34.3)	271 (55.0)	
**Race, *n* (%)**				
Han ethnicity	120 (13.9)	358 (41.4)	387 (44.7)	0.172
Minority ethnicity	2 (10.0)	6 (30.0)	12 (60.0)	
**Smoking status, *n* (%)**				
Never	113 (13.3)	348 (41.0)	388 (45.7)	0.252
Occasionally	7 (25.9)	12 (44.4)	8 (29.6)	
Often	2 (22.2)	4 (44.4)	3 (33.3)	
**Alcohol consumption, *n* (%)**				
Never	3 (37.5)	4 (50.0)	1 (12.5)	0.002**
Occasionally	62 (17.6)	151 (42.8)	140 (39.7)	
Often	57 (10.9)	209 (39.9)	258 (49.2)	
**Exercise duration, *n* (%)**				
0–1 h	71 (10.5)	287 (42.3)	321 (47.3)	< 0.001***
2–3 h	31 (15.4)	111 (55.2)	59 (29.4)	
≥ 4 h	3 (60)	1 (20.0)	1 (20.0)	
**Sedentariness, *n* (%)**				
1–3 h	4 (7.3)	27 (49.1)	24 (43.6)	0.151
4–7 h	80 (16.0)	203 (40.7)	216 (43.3)	
≥ 8 h	38 (11.5)	134 (40.5)	159 (48.0)	
**Mobile phone usage time, *n* (%)**				
1–3 h	18 (13.6)	66 (50.0)	48 (36.4)	0.154
4–7 h	65 (12.9)	203 (40.4)	234 (46.6)	
≥ 8 h	39 (15.5)	95 (37.8)	117 (46.6)	
**Sleep duration, *n* (%)**				
1–6 h	25 (15.0)	69 (41.3)	73 (43.7)	0.443
7–8 h	86 (13.8)	263 (42.1)	275 (44.1)	
≥ 9 h	11 (11.7)	32 (34.0)	51 (54.3)	
**Breakfast consumption, *n* (%)**				
Never	7 (10.1)	29 (42.0)	33 (47.8)	0.869
Occasionally	54 (13.4)	165 (40.9)	184 (45.7)	
Often	61 (14.8)	170 (41.2)	182 (44.1)	
**Midnight snack, *n* (%)**				
Never	40 (13.5)	125 (42.2)	131 (44.3)	0.349
Occasionally	64 (13.0)	195 (39.7)	232 (47.3)	
Often	18 (18.4)	44 (44.9)	36 (36.7)	
**Water consumption, *n* (%)**				
< 1 L	23 (10.1)	76 (33.5)	128 (56.4)	< 0.01**
1–2 L	84 (14.8)	249 (43.9)	234 (41.3)	
> 2 L	15 (16.5)	39 (42.9)	37 (40.7)	
**Takeout frequency, *n* (%)**				
Never	28 (17.0)	74 (44.8)	63 (38.2)	0.299
Occasionally	59 (12.6)	193 (41.2)	216 (46.2)	
Often	35 (13.9)	97 (38.5)	120 (47.6)	
**Nutrition knowledge, *n* (%)**				
Poor	29 (16.3)	71 (39.9)	78 (43.8)	0.728
Generally	73 (13.7)	216 (40.7)	242 (45.6)	
Good	20 (11.4)	77 (43.8)	79 (44.9)	

**p < 0.01, ***p < 0.001, PBF, percentage of body fat; PBF for males: < 14% is low, 14%–25% is normal, and > 25% is high; for females: < 17% is low, 17%–30% is normal, and > 30% is high.

### 3.2 Body composition analysis by percentage of body fat classification

To further understand the effect of obesity on body composition, we performed the analysis in Supplementary Table 1. The analysis showed that participants with a lower body fat percentage had higher levels of water, protein, inorganic salts, and skeletal muscle. Furthermore, significant differences were observed when obesity was defined by other indicators (*p* < 0.01).

### 3.3 Comparison of different obesity indicators

**FIGURE 1:**
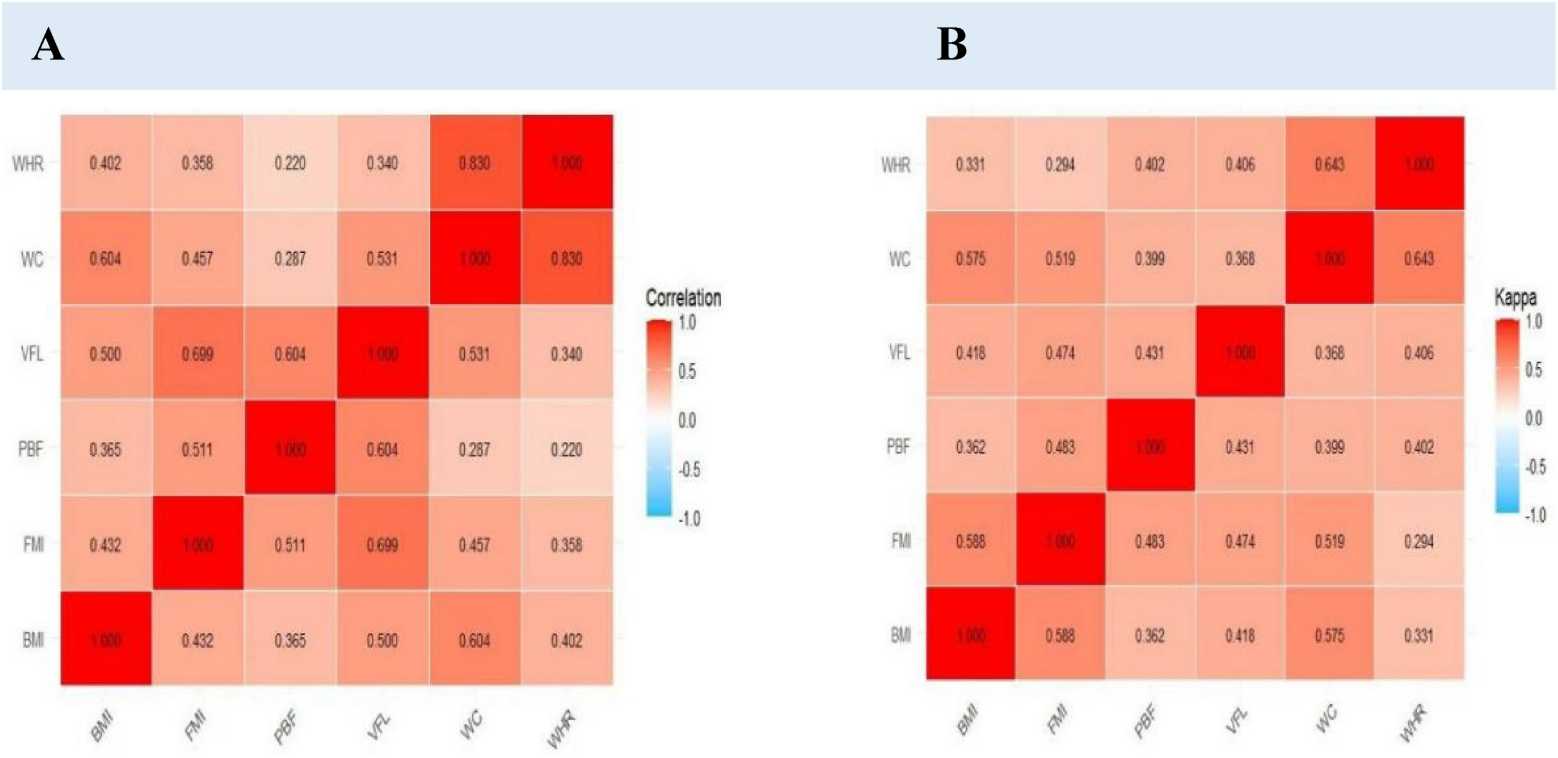
Correlation and kappa concordance of obesity indicators in college students. **(A)** Correlation of obesity indicators. **(B)** Kappa concordance of obesity indicators.

The prevalence of obesity and overweight varies significantly depending on the indicators employed. These metrics include BMI, FMI, PBF, VFL, WC, and WHR. From Supplementary Table 2, PBF identified the highest proportion of individuals classified as overweight and obese (42.5%), followed by WC (40.2%), FMI (37.1%), and BMI (30.7%). Although more men than women were categorized as overweight or obese across all indicators, none of these differences were statistically significant. Since the detection rates of obesity and overweight vary depending on the indicators used, we conducted a comparison of different obesity indicators, as shown in Figure 1. The correlation and consistency across different obesity metrics are not uniform; however, they exhibit low levels of agreement.

### 3.4 Construction of dietary patterns for research subjects

**FIGURE 2:**
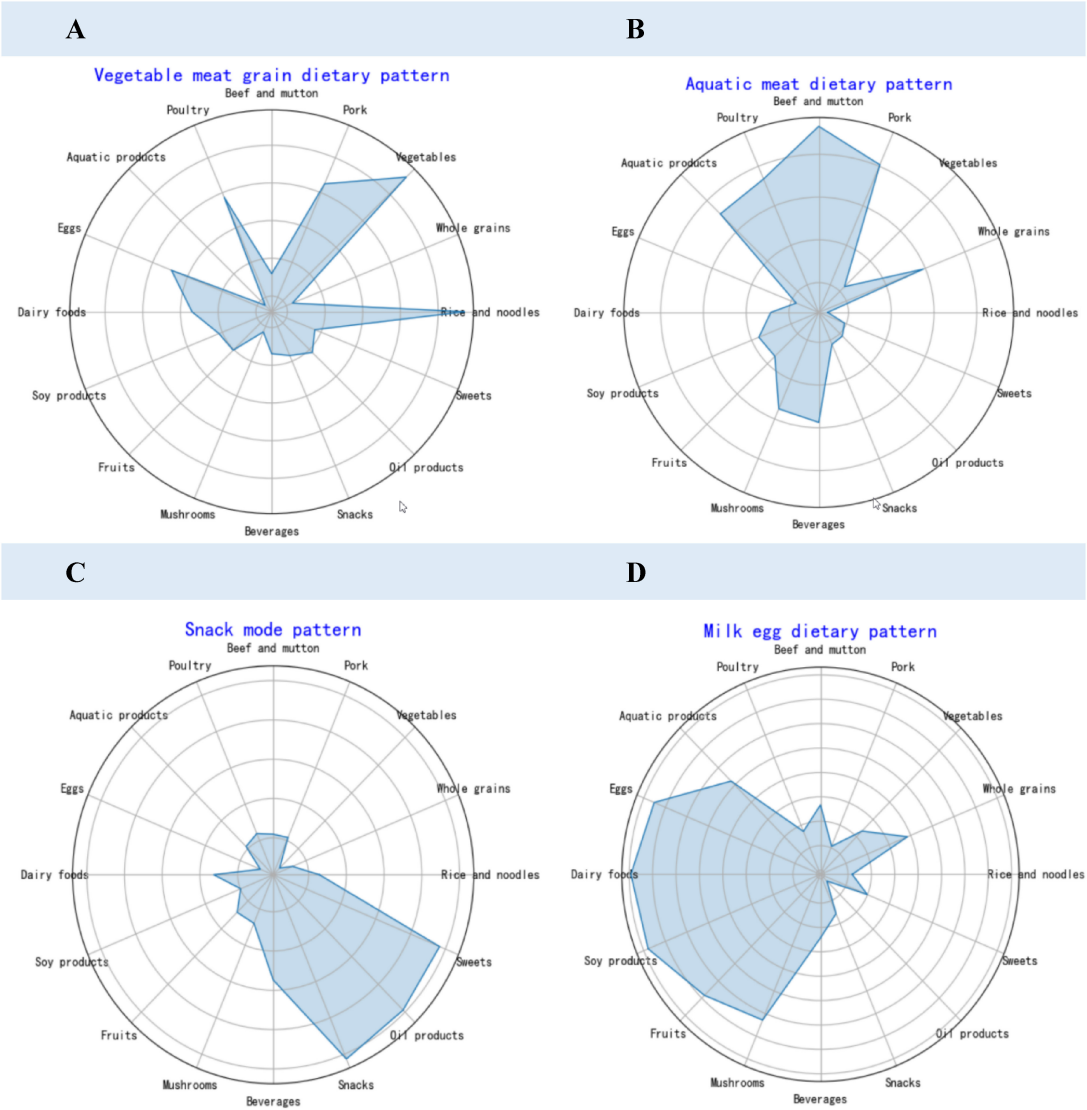
Dietary pattern factor loading radar chart. **(A)** Vegetable meat grain dietary pattern. **(B)** Aquatic meat dietary pattern. **(C)** Snack mode pattern. **(D)** Milk egg dietary pattern.

In order to gain a more comprehensive understanding of the impact of diet on obesity, we conducted a dietary pattern analysis, as shown in Figure 2. Dietary patterns were identified using principal component analysis (PCA). From Supplementary Tables 3, 4, Supplementary Figure 1, Suitability verification results showed a KMO (Kaiser–Meyer–Olkin) value of 0.84 (> 0.7) and Bartlett’s sphericity test *P* < 0.001, indicating that the dietary data were appropriate for factor analysis. Four main factors were extracted, accounting for a cumulative contribution rate of 50.0%. After applying maximum variance orthogonal rotation, dietary patterns were named based on factor loadings with absolute values greater than 0.4.

The first factor, vegetable meat grain dietary pattern, consisted of rice, grains, vegetables, bean products, fruits, and mushrooms. The second factor, aquatic meat dietary pattern, was characterized by pork, beef, mutton, poultry, aquatic products, and oil products. The third factor, Snack mode dietary pattern, included beverages, snacks, and sweets. The fourth factor, Milk egg dietary pattern, was primarily composed of dairy products.

### 3.5 Relationship between dietary patterns and obesity in college students

In order to clarify the relationship between dietary patterns and a variety of different obesities, a series of analysis were performed as follows. The research results from Table 2 indicate that all four dietary patterns were associated with PBF. Aquatic meat dietary pattern and Snack mode dietary pattern were linked to an increased risk of PBF, while the vegetable meat grain dietary pattern and milk egg dietary pattern were associated with a decreased risk of PBF (*P* < 0.01). Furthermore, it was found that aquatic meat dietary pattern increases the risk of FMI (*P* < 0.05) and VFL (*P* < 0.01) from Supplementary Tables 6–10, while Snack mode dietary pattern increased the risk of WHR (adjusted before *P* < 0.05) and WC (*P* < 0.05).

**TABLE 2:** Logistic regression analysis of the relationship between dietary patterns and percentage of body fat (PBF) in college students.

Dietary patterns	Dietary pattern score grouping			*P*
	T1	T2	T3	
**Vegetable meat grain dietary pattern**				
Model I	1.00 (ref)	1.58 (1.14, 2.19)	1.06 (0.76, 1.47)	< 0.006**
Model II	1.00 (ref)	1.58 (1.14, 2.19)	1.07 (0.77, 1.49)	< 0.006**
**Aquatic meat dietary pattern**				
Model I	1.00 (ref)	0.64 (0.46, 0.89)	0.92 (0.67, 1.27)	< 0.008**
Model II	1.00 (ref)	0.64 (0.46, 0.89)	0.92 (0.66, 1.27)	< 0.008**
**Snack mode dietary pattern**				
Model I	1.00 (ref)	0.68 (0.49, 0.94)	0.80 (0.58, 1.11)	< 0.020*
Model II	1.00 (ref)	0.68 (0.49, 0.94)	0.80 (0.58, 1.11)	< 0.019*
**Milk egg dietary pattern**				
Model I	1.00 (ref)	1.70 (1.22, 2.35)	1.17 (0.84, 1.63)	< 0.002**
Model II	1.00 (ref)	1.68 (1.21, 2.34)	1.17 (0.84, 1.63)	< 0.002**

*p < 0.05, **p < 0.01. Model I (was not adjusted), Model II (adjusted for demographic factors).

### 3.6 Development and validation of a dietary pattern-based PBF risk model

#### 3.6.1 Basic characteristics of the training set and validation set

For better predictive modeling analysis and validation, a total of 885 research subjects were randomly divided as 7:3, with 620 subjects comprising the training set and 265 subjects forming the validation set. The research findings revealed that the obesity prevalence of PBF in the training set was 42.1%, while it was 43.4% in the validation set. However, this difference was not statistically significant (*P* = 0.73). As shown in Table 3.

**TABLE 3:** The basic information of the training set and validation set for the research.

Variables	Validation set	Training set	χ^2^	*P*
**PBF, *n* (%)**				
Yes	115 (43.4)	261 (42.1)	0.128	0.73
No	150 (56.6)	359 (57.9)		
**Gender, *n* (%)**				
Males	152 (57.4)	341 (55.0)	0.419	0.518
Females	113 (42.6)	279 (45.0)		
**Age, *n* (%)**				
≤ 20	62 (23.4)	285 (46.0)	39.682	< 0.001***
> 20	203 (76.6)	225 (54.0)		
**Income, *n* (%)**				
Low	183 (69.1)	417 (67.3)	0.275	0.6
High	82 (30.9)	203 (32.7)		
**Smoking status, *n* (%)**				
No	10 (3.8)	26 (4.2)	0.084	0.772
Yes	255 (96.2)	594 (95.8)		
**Alcohol consumption, *n* (%)**				
No	115 (43.4)	246 (39.7)	1.063	0.303
Yes	150 (56.5)	374 (60.3)		
**Physical exercise, *n* (%)**				
No	56 (21.1)	150 (24.2)	0.974	0.324
Yes	209 (78.9)	470 (75.8)		
**Prolonged sitting, *n* (%)**				
No	251 (94.7)	579 (93.4)	0.563	0.453
Yes	14 (5.3)	41 (6.6)		
**Mobile phone usage, *n* (%)**				
< 3 h	33 (12.5)	99 (16.0)	1.807	0.179
≥ 3 h	232 (87.5)	521 (84.0)		
**Length of sleep, *n* (%)**				
< 6 h	47 (17.7)	120 (19.4)	0.318	0.573
≥ 6 h	218 (82.3)	500 (80.6)		
**Quality of sleep, *n* (%)**				
No	7 (2.6)	24 (3.9)	0.83	0.362
Yes	258 (97.4)	596 (96.1)		

***p < 0.001.

#### 3.6.2 Construction and validation of a PBF obesity risk assessment model for college students based on dietary patterns

From Supplementary Table 5, it further illustrates the impact of healthy diet extraction of PBF. Population demographics, lifestyle, dietary habits, and healthy diet extraction were used as independent variables, and whether or not a person had PBF obesity was used as the dependent variable to enter the model. Predictive factors were selected, and a PBF obesity risk assessment model was constructed. The research results showed that the predictive factors in the model in the training set mainly included gender, exercise, late-night snacking, regular meals, and healthy diet extraction. The results of the relevant regression analysis were shown in Table 4.

**TABLE 4:** PBF obesity risk assessment model for college students in the training set.

Variables	β	OR	95% CI		*P*
			Lower	Upper	
Gender	−0.627	0.534	0.362	0.789	< 0.002**
Exercise	−0.657	0.518	0.325	0.826	0.006**
Late-night snacking	2.286	9.835	6.526	14.821	< 0.001***
Regular meal patterns	1.519	4.566	2.143	9.729	< 0.001***
Healthy dietary extracts	−0.040	0.961	0.938	0.985	< 0.002**
Constant	−2.610	0.074	–	–	< 0.001***

**p < 0.01, ***p < 0.001.

**FIGURE 3:**
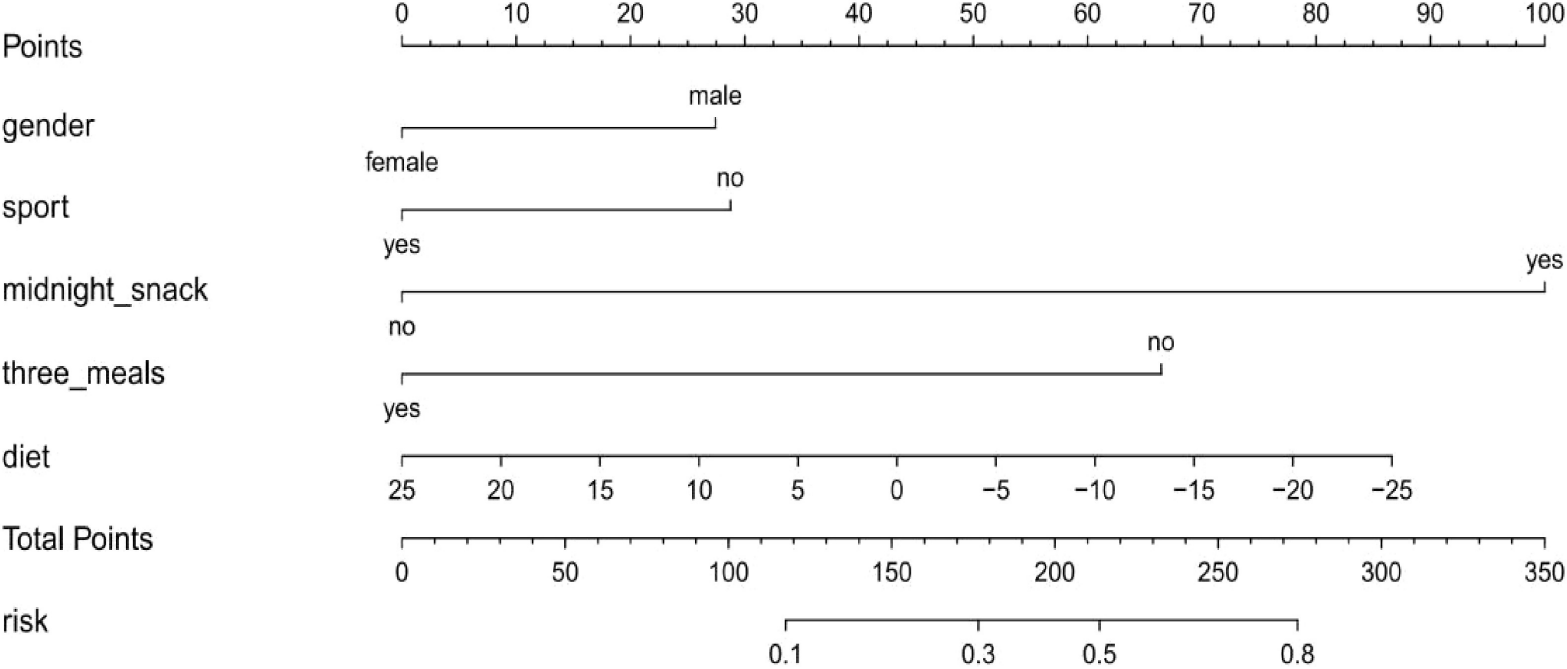
Nomogram for the PBF obesity risk assessment model based on a training set of college students.

A line chart depicting the risk assessment model constructed from the training set can be used to calculate the total score based on the values of each predictive factor, thereby evaluating the probability of developing PBF Obesity. Specific results were presented in Figure 3.

**FIGURE 4:**
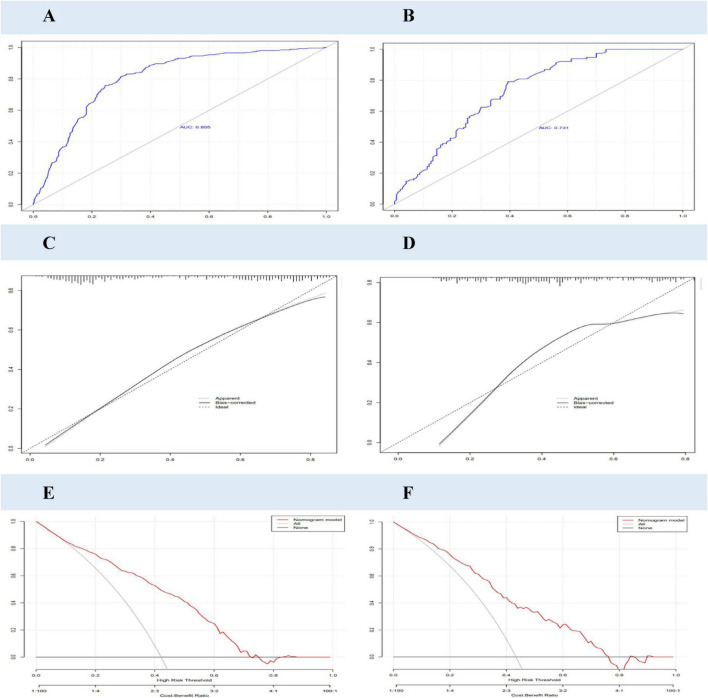
Adaption of the PBF obesity risk assessment model of college students. **(A,B)** OC curves of PBF obesity risk model for college students in training and validation sets; **(C,D)** calibration curves of PBF obesity risk model for college students in training and validation sets; **(E,F)** DCA curves of PBF obesity risk model for college students in training and validation sets.

The PBF obesity risk assessment model underwent rigorous internal validation, demonstrating robust predictive performance, as illustrated in Figure 4. The model exhibited excellent discriminatory ability, with an AUC of 0.805 in the training set and 0.731 in the validation set. Calibration accuracy, assessed using the Hosmer–Lemeshow test, was high in both the training and validation sets (*P* > 0.05), indicating strong agreement between predicted and observed outcomes. Furthermore, clinical DCA revealed that the model provides significant clinical utility, with its curves substantially surpassing the “treat all” and “treat none” reference lines, underscoring its net clinical benefit. Collectively, these findings affirm the reliability and clinical applicability of the PBF obesity risk assessment model for identifying individuals at risk of obesity.

## 4 Discussion

The investigation revealed a lack of research on risk assessment for overweight and obesity in college students based on dietary patterns. Our study found that the prevalence of overweight and obesity among college students is relatively high and overall obesity prevalence analysis revealed 42.5% of participants (376 out of 885) had PBF-defined obesity. PBF-defined obesity was defined as > 25% body fat for males and > 30% for females; significantly more males were affected (55.0%) than females (32.7%). Supplementary Table 1 demonstrates varying results across the six obesity indicators, further highlighting the differences between these metrics. A comprehensive analysis incorporating multiple indicators can help reduce measurement errors. The “vegetable meat grain dietary pattern” and “milk egg dietary pattern” were associated with a reduced risk of PBF, while the “snack mode dietary pattern” and “aquatic meat dietary pattern” dietary patterns were linked to an increased risk of PBF. Binary logistic regression analysis identified five key predictors of body fat percentage obesity in college students: gender, exercise habits, late-night snacking, regular meal patterns, and adherence to a healthy diet.

The observed associations between dietary patterns and obesity phenotypes were mediated through distinct metabolic mechanisms, where the meat and aquatic product dietary pattern—rich in saturated and monounsaturated fats—upregulated *de novo* lipogenesis via SREBP-1c activation, promoted visceral adiposity and hepatic insulin resistance that reduced Very Low-Density Lipoprotein clearance (29); conversely, the vegetable meat grain pattern provided polyunsaturated fatty acids (PUFAs) activating PPAR-α pathways to enhance β-oxidation and reduce FMI and VFL. The Snack mode pattern’s high sugar load induced postprandial hyperinsulinemia, promoting adipocyte differentiation through PI3K or Akt signaling. Fructose metabolism specifically stimulated hepatic *de novo* lipogenesis via ChREBP activation (30). The milk egg pattern delivered bioactive peptides that increased thermogenesis through UCP1 upregulation in brown adipose tissue, and its calcium content inhibited calcitriol-mediated adipocyte Ca^2+^ influx to suppress lipogenesis. Additionally, plant-based fibers in the vegetable pattern were fermented to SCFAs, such as butyrate, which improved insulin sensitivity via GLP-1 secretion and AMPK activation, explaining its protective effect against PBF accumulation (31).

The prevalence of overweight and obesity among college students has become a growing concern. According to the BMI evaluation standard, 30.7% of students are classified as overweight or obese. However, BMI may underestimate the true prevalence of obesity (15). When assessed using the body fat percentage standard, the prevalence increases to 42.5%, reflecting improved predictive accuracy (32). This perspective was supported by other studies (33). This situation can be attributed to the heavy academic pressure faced by young students and the widespread use of electronic devices, which contribute to sedentary behavior, insufficient physical activity, and unhealthy dietary habits. These factors collectively led to abdominal fat accumulation and relatively low muscle mass (33).

Abdominal fat is commonly assessed using indices such as VFL, WHR, and WC. In our study, the prevalence of central obesity varied significantly depending on the measure used: 21.0% based on the VFL standard, 35.6% using WHR, and 40.2% using WC. The correlation and consistency among these measures were not statistically significant, likely due to their inherent strengths and limitations, as well as variations in body composition, muscle mass, and gender-related fat distribution (34).

This study also explored the relationship between dietary patterns and various obesity indicators. The first dietary pattern, characterized by a traditional Chinese plant-based diet rich in rice, wheat, whole grains, vegetables, bean products, fruits, and mushrooms, was negatively associated with BMI and PBF. These findings aligned with previous research suggesting that plant-based diets, similar to the Eastern dietary pattern, were linked to a lower risk of obesity (35). Furthermore, evidence indicated that higher vegetable consumption was negatively associated with body weight, while fruit and vegetable intake is positively associated with a lower body fat percentage (36).

In contrast, the second dietary pattern, primarily composed of pork, poultry, livestock, aquatic products, and oil-based foods, showed positive associations with PBF, FMI, and VFL. This pattern is closer to a Western dietary style and is more prevalent among students from high-income households. Due to its higher fat content compared to other patterns, it is more likely to contribute to abdominal obesity, as evidenced by elevated FMI and VFL. These results were consistent with previous studies linking high-fat diets to increased risks of central obesity (37).

Research indicates that dietary patterns rich in saturated and monounsaturated fatty acids are associated with a 25% increase in the odds of developing metabolic disorders. A study of Filipino adults aged 20 and above found that a dietary pattern primarily consisting of meat and sugary drinks was significantly positively associated with diabetes, overweight, and obesity (38).

The third dietary pattern, characterized by a high intake of beverages, snacks, and sweets, represents a common junk food diet among students. It is positively correlated with PBF, WHR, and WC. This pattern’s high proportion of sugary drinks aligns with evidence showing that unhealthy foods, including sweets and sugary beverages, may increased the risk of abdominal obesity (39). Sugar intake, in particular, has been strongly linked to visceral fat accumulation and other body composition variables (40).

In contrast, the fourth dietary pattern, dominated by dairy and egg products, was negatively associated with PBF and BMI. Dairy products are rich in essential vitamins and minerals, while eggs provide high-quality protein and calcium. Adherence to this pattern ensured sufficient nutrient intake and reduced the risk of obesity (41).

To further investigate, a simplified healthy dietary pattern score was developed using posterior inference to extract dietary patterns. This approach has been widely applied in studies examining the relationship between diet and metabolic diseases such as non-alcoholic fatty liver disease, hypertension, and hyperlipidemia (42). Based on the four identified dietary patterns, a simple dietary score was constructed and found to be significantly correlated with PBF. The food items included in the score reflect the eating habits of the study population and emphasize food groups with a notable impact on obesity. This healthy dietary pattern showed a stronger association with PBF risk, thereby enhancing the potential for targeted obesity prevention strategies.

Our study identified several key factors influencing overweight and obesity among college students, including gender, physical activity, sleep duration, fluid intake, alcohol consumption, regular meals, and adherence to a healthy diet. The prevalence of PBF-defined obesity among male students was 55.0%, significantly higher than the 32.7% observed in females. Other obesity indicators also revealed higher prevalence rates among males, consistent with global and domestic trends (43). This disparity can largely be attributed to lifestyle differences: males are more likely to engage in smoking and alcohol consumption, whereas females tend to adopt healthier habits, such as higher fruit and vegetable intake, reduced alcohol and meat consumption, and increased physical activity (44).

Alcohol consumption was confirmed as a significant risk factor for overweight and obesity among college students. A prospective study demonstrated that reducing weekly alcohol intake and avoiding excessive drinking can help control waist circumference and BMI in men (45). Excessive and frequent alcohol consumption during youth has been shown to contribute to weight gain (46).

Physical activity emerged as one of the most effective ways to improve obesity-related metrics. Increasing moderate-intensity exercise while reducing low-intensity activities significantly improved obesity indicators (47). Additionally, more physical activity, less screen time, and sufficient sleep were positively associated with lower BMI, reduced cardiometabolic risk, and improved cognitive development, such as enhanced motor skills and academic performance (48).

Sleep duration also played a crucial role. A study of Chinese adolescents found that short sleep duration was associated with increased overweight or obesity, particularly central obesity in boys (49). Similarly, adequate hydration has been linked to healthier body composition. Research suggested that sufficient water intake might help prevent abdominal obesity, particularly in young adults (50).

Moreover, higher meal frequency was associated with slightly lower body fat percentage (51). Furthermore, a higher eating frequency could be associated with diet quality improvement, lower adiposity, and lower risk of developing MetS or its components (52).

Finally, our study developed a non-invasive obesity risk assessment model using PBF as the primary indicator, incorporating demographic, dietary, and lifestyle factors. The use of the InBody 570 analyzer provided accurate body composition data within a short timeframe (53), and its reliability has led to frequent application in obesity research (54). The resulting model demonstrated strong discriminatory power and calibration, with an AUC exceeding 0.7. In addition to traditional measures such as BMI and WC for defining overall and abdominal obesity, this study included comprehensive body composition indicators such as FMI, VFL, WHR, and PBF. While our PBF-based nomogram (AUC = 0.805) demonstrates robust predictive performance for college-specific obesity screening, we acknowledge the need for contextualizing these findings against existing models. In direct comparison to Liu et al. (24)—whose machine learning model predicted BMI-defined obesity in Chinese adolescents with an AUC of 0.72—our approach achieves key advancements through outcome specificity. We target PBF-defined obesity, a more sensitive adiposity indicator than BMI, capturing a 42.5% prevalence rate versus BMI’s 30.7% prevalence. Behavioral granularity emerges when incorporating dietary pattern scores such as the healthy diet extract (OR = 0.961, *p* < 0.002), enabling precise behavioral interventions, whereas Liu et al.’s (24) model relied primarily on demographic or anthropometric factors. Visualizing the results through line charts enhances the model’s practical utility, making it a valuable tool for college health programs. However, the model’s applicability may be limited to the local population, necessitating further validation in diverse cohorts.

Despite its contributions, this study has several limitations. First, as a cross-sectional survey, it cannot establish causal relationships or determine temporal sequences. Second, the sample was limited to college students from a specific region, which limits the generalizability of the findings. Third, the lack of parental body shape data in the questionnaire design prevented the inclusion of hereditary factors in the regression analysis. Future research should focus on larger and more diverse samples, employ longitudinal designs, and explore genetic influences on body composition to enhance the predictive accuracy of obesity risk models. Additionally, validating the model in external populations is crucial to improve its applicability beyond the local context.

## 5 Conclusion

In this study, the non-invasive college body fat obesity risk assessment model, combined with the ROC curve, offers valuable support for the primary prevention and management of obesity in college students. Furthermore, we found a significant association between dietary patterns and obesity among college students. Four distinct dietary patterns were identified: the vegetable meat grain dietary pattern, the aquatic meat dietary pattern, the snack mode dietary pattern and the milk egg dietary pattern. A healthy dietary pattern, constructed based on these four patterns, was negatively associated with obesity risk in college students. Binary logistic regression analysis highlighted four key predictors of body fat percentage obesity: gender, physical activity, late-night snacking, regular meals, and adherence to a healthy diet.

## Data Availability

The original contributions presented in this study are included in this article/Supplementary material, further inquiries can be directed to the corresponding author.
