# Supplementary material for: Risk factors analysis and prediction models of obesity in college students based on dietary patterns
Source: Front Nutr. 2025 Sep 11;12:1598946. doi: 10.3389/fnut.2025.1598946 (PMC12460112; doi:10.3389/fnut.2025.1598946)
Supplement: Supplementary file 1 [file Data_Sheet_1.pdf]

Risk Factors Analysis and Prediction Models of Obesity in College Students Based on Dietary Patterns

Supplementary Materials

Figure S1. KMO and Bartlett's Test..2

Table S1. Body Composition Analysis Stratified by Percentage of Body Fat....................2

Table S2. The proportion of obesity among college students measured by different indicator 2

Table S3. KMO and Bartlett's Test..3

Table S4. Rotated Component Matrixa..3

Table S5. Logistic regression analysis was conducted to assess the association between healthy dietary patterns and PBF in college students..4

Table S6. Logistic regression analysis of the relationship between dietary patterns and Body Mass Index(BMI) in college students 4

Table S7. Logistic regression analysis of the relationship between dietary patterns and Fat Mass Index(FMI) in college students 5

Table S8. Logistic regression analysis of the relationship between dietary patterns and Visceral Fat Leve(VFL) in college students 5

Table S9. Logistic regression analysis of the relationship between dietary patterns and Waist-to-Hip Ratio(WHR) in college students 6

Table S10. Logistic regression analysis of the relationship between dietary patterns and Waist Circumference(WC) in college students 6

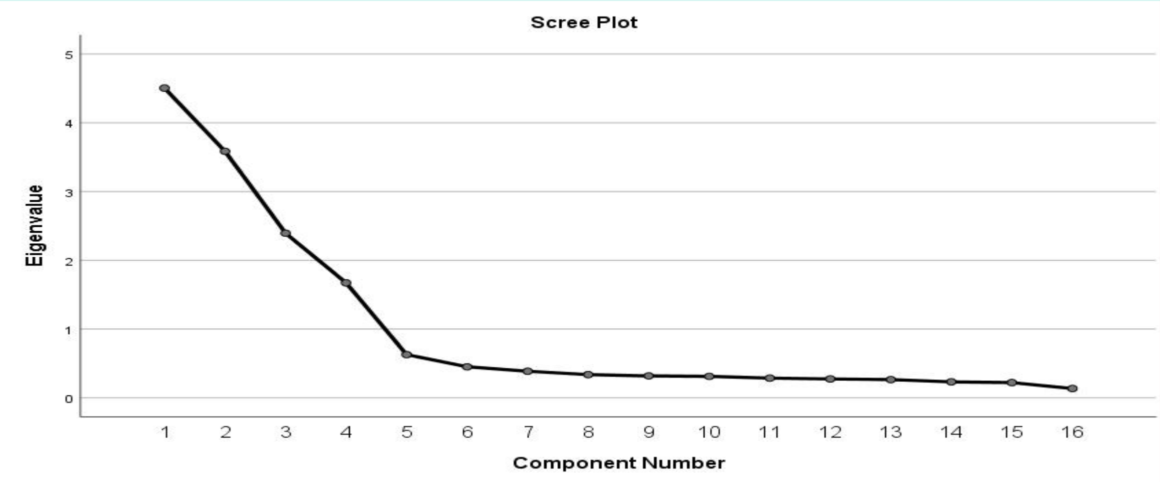

FIGURE S1. Scree Plot

TABLE S1. Body Composition Analysis Stratified by Percentage of Body Fat

Variables	PBF(%)			***P***
	Low	Normal	High	
TBW（L）	40.01±5.81	37.03±7.77	34.13±8.09	**<0.001*****
Protein（Kg）	10.80±1.58	9.97±2.15	9.14±2.23	**<0.001*****
Minerals（Kg）	3.74±0.58	3.60±0.73	3.39±0.76	**<0.001*****
SMM（Kg）	30.59±4.79	28.08±6.48	25.59±6.71	**<0.001*****
SMI（Kg/m^2）	7.58±0.80	7.10±1.15	6.77±1.23	**<0.001*****
InBody Score	72.91±6.66	73.45±4.38	68.49±5.54	**<0.001*****

Note: p<0.001***, PBF: Percentage of Body Fat, TBW: Total Body Water, SMM: Skeletal Muscle Mass, SMI: Skeletal Muscle Index.

TABLE S2. The proportion of obesity among college students measured by different indicators

**Variables**	**Total**	**Female**	**Male**	χ^2	***P***
BMI, *n(%)*	272(30.7)	130(14.7)	142(16.0)	1.950	0.163
FMI, *n(%)*	328(37.1)	150(16.9)	178(20.1)	0.437	0.509
PBF, *n(%)*	376(42.5)	174(19.7)	202(22.8)	1.042	0.307
VFL, *n(%)*	186(21.0)	80(9.0)	106(12.0)	0.157	0.692
WHR, *n(%)*	315(35.6)	135(15.3)	180(20.3)	0.409	0.522
WC, *n(%)*	356(40.2)	163(18.4)	193(21.8)	0.538	0.463

Note: PBF: Percentage Body Fat, BMI: Body Mass Index, VFL: Visceral Fat Leve, WHR: Waist-to-Hip Ratio, WC: Waist Circumference, FMI: Fat Mass Index.

TABLE S3. KMO and Bartlett's Test		
Kaiser-Meyer-Olkin Measure of Sampling Adequacy.		0.846
Bartlett's Test of Sphericity	Approx. Chi-Square	12941.118
	df	120
	Sig.	0.000

TABLE S4. Rotated Component Matrixa				
**Component**	**Factor**			
	1	2	3	4
Rice and wheat	.018	.057	-.098	.739
Whole grains	.285	-.077	.391	-.164
Vegetables	.133	-.140	.031	.727
Pork	.007	.017	.608	.451
Beef and mutton	.167	.018	.731	-.082
Poultry	.073	.039	.536	.374
Aquatic products	.423	.015	.512	-.235
Eggs	.652	-.114	-.025	.292
Dairy products	.693	.127	.085	.139
Soy products	.680	-.002	.164	.019
Fruits	.584	.082	.151	.005
Edible fungi	.529	.079	.349	-.168
Beverages	.143	.347	.375	-.061
Fried foods	-.079	.786	.017	.022
Snacks	.057	.827	.022	-.032
Pastries	.099	.767	-.009	-.035

TABLE S5. Logistic regression analysis was conducted to assess the association between healthy dietary patterns and PBF in college students

Healthy dietary pattern	Dietary pattern score grouping			*P*
	T1	T2	T3	
Model I	1.00	0.97(0.82,1.14)	0.85(0.72,1.01)*	0.001**
Model II	1.00	0.96(0.81,1.14)	0.84(0.71,1.00)*	0.001**

Note: p <0.05*, p<0.01** Model I was not adjusted, Model II adjusted for demographic factors.

TABLE S6. Logistic regression analysis of the relationship between dietary patterns and Body Mass Index(BMI) in college students

Dietary patterns	Dietary Pattern Score Grouping			*P*
	T1	T2	T3	
Vegetable meat grain dietary pattern				
Model I	1.00	1.48(1.04,2.11)	1.06(0.97,1.99)	0.031*
Model II	1.00	1.48(1.04,2.12)	1.41(0.98,2.01)	0.030*
Aquatic meat dietary pattern				
Model I	1.00	0.76(0.53,1.08)	1.02(0.72,1.43)	0.126
Model II	1.00	0.76(0.53,1.08)	1.00(0.71,1.42)	0.130
Snack mode dietary pattern				
Model I	1.00	0.82(0.58,1.17)	0.99(0.70,1.39)	0.282
Model II	1.00	0.82(0.58,1.17)	0.99(0.70,1.40)	0.273
Milk egg dietary pattern				
Model I	1.00	1.62(1.15,2.32)	1.18(0.83,1.70)	0.006**
Model II	1.00	1.61(1.13,2.29)	1.18(0.83,1.70)	0.008**

Note: p <0.05*, p<0.01** Model I was not adjusted, Model II adjusted for demographic factors.

TABLE S7. Logistic regression analysis of the relationship between dietary patterns and Fat Mass Index(FMI) in college students

Dietary patterns	Dietary Pattern Score Grouping			*P*
	T1	T2	T3	
Vegetable meat grain dietary pattern				
Model I	1.00	1.25(0.89,1.74)	1.21(0.87,1.70)	0.200
Model II	1.00	1.25(0.89,1.75)	1.22(0.87,1.71)	0.198
Aquatic meat dietary pattern				
Model I	1.00	0.65(0.47,0.91)	0.92(0.66,1.28)	0.013*
Model II	1.00	0.65(0.47,0.92)	0.92(0.66,1.27)	0.013*
Snack mode dietary pattern				
Model I	1.00	1.23(0.88,1.71)	1.13(0.80,1.58)	0.233
Model II	1.00	1.22(0.88,1.71)	1.13(0.80,1.58)	0.236
Milk egg dietary pattern				
Model I	1.00	1.34(0.96,1.70)	1.13(0.80,1.58)	0.089
Model II	1.00	1.33(0.95,1.86)	1.13(0.80,1.58)	0.097

Note: $p<0.05^{*}$, $p<0.01^{**}$ Model I was not adjusted, Model II adjusted for demographic factors.

TABLE S8. Logistic regression analysis of the relationship between dietary patterns and Visceral Fat Leve(VFL) in college students

Dietary patterns	Dietary Pattern Score Grouping			*P*
	T1	T2	T3	
Vegetable meat grain dietary pattern				
Model I	1.00	1.18(0.79,1.75)	1.09(0.73,1.62)	0.419
Model II	1.00	1.18(0.79,1.75)	1.08(0.73,1.62)	0.421
Aquatic meat dietary pattern				
Model I	1.00	0.51(0.33,0.76)	0.83(0.57,1.21)	0.001*
Model II	1.00	0.50(0.33,0.76)	0.83(0.57,1.22)	0.001*
Snack mode dietary pattern				
Model I	1.00	1.00(0.67,1.49)	1.00(0.67,1.49)	0.999
Model II	1.00	1.00(0.67,1.49)	1.00(0.67,1.48)	0.996
Milk egg dietary pattern				
Model I	1.00	1.00(0.67,1.48)	0.94(0.63,1.40)	0.761
Model II	1.00	1.01(0.68,1.49)	0.94(0.63,1.40)	0.762

Note: $p<0.05^{*}$, $p<0.01^{**}$ Model I was not adjusted, Model II adjusted for demographic factors.

TABLE S9. Logistic regression analysis of the relationship between dietary patterns and Waist-to-Hip Ratio(WHR) in college students

Dietary patterns	Dietary Pattern Score Grouping			*P*
	T1	T2	T3	
Vegetable meat grain dietary pattern				
Model I	1.00	1.06(0.76,1.48)	0.90(0.64,1.26)	0.545
Model II	1.00	1.06(0.76,1.48)	0.90(0.64,1.26)	0.526
Aquatic meat dietary pattern				
Model I	1.00	0.86(0.61,1.21)	1.06(0.76,1.48)	0.387
Model II	1.00	0.86(0.61,1.21)	1.07(0.76,1.49)	0.381
Snack mode dietary pattern				
Model I	1.00	0.51(0.36,0.72)	0.78(0.56,1.08)	0.000**
Model II	1.00	0.51(0.36,0.72)	0.78(0.56,1.08)	0.996
Milk egg dietary pattern				
Model I	1.00	1.09(0.78,1.53)	1.09(0.78,1.53)	0.605
Model II	1.00	1.10(0.79,1.55)	1.09(0.78,1.53)	0.574

Note: $p<0.05^{*}$, $p<0.01^{**}$ Model I was not adjusted, Model II adjusted for demographic factors.

TABLE S10. Logistic regression analysis of the relationship between dietary patterns and Waist Circumference(WC) in college students

Dietary patterns	Dietary Pattern Score Grouping			*P*
	T1	T2	T3	
Vegetable meat grain dietary pattern				
Model I	1.00	1.22(0.88,1.70)	1.29(0.93,1.80)	0.130
Model II	1.00	1.22(0.88,1.70)	1.30(0.93,1.81)	0.122
Aquatic meat dietary pattern				
Model I	1.00	0.99(0.71,1.37)	1.00(0.72,1.39)	0.933
Model II	1.00	0.99(0.71,1.37)	1.00(0.72,1.38)	0.941
Snack mode dietary pattern				
Model I	1.00	0.70(0.50,0.98)	0.88(0.64,1.22)	0.036*
Model II	1.00	0.70(0.50,0.98)	0.88(0.64,1.23)	0.035*
Milk egg dietary pattern				
Model I	1.00	1.34(0.97,1.87)	1.03(0.74,1.44)	0.079
Model II	1.00	1.33(0.96,1.85)	1.03(0.74,1.44)	0.087

Note: $p<0.05^{*}$, $p<0.01^{**}$ Model I was not adjusted, Model II adjusted for demographic factors.
